# Myocardial vasculature: the third significant contributor to MR diffusion signals in the isolated rabbit heart

**DOI:** 10.1186/1532-429X-11-S1-P42

**Published:** 2009-01-28

**Authors:** Min-Sig Hwang, Melvin Clark, John R Forder

**Affiliations:** Mcknight Brain Institute, University of Florida, Gainesville, FL USA

**Keywords:** Apparent Diffusion Coefficient, Slow Component, Free Wall, Vascular Space, Vascular Compartment

## Introduction

The coronary circulation is a significant component of the myocardial mass, due to the high energetic demand of cardiac tissue – the capillary surface area in the heart occupies 14–22% by volume [1]. Thus, vascular spaces need to be considered in addition to interstitial and intracellular spaces for a comprehensive understanding of the physiological compartments responsible for the diffusion signals measured by magnetic resonance. Understanding the origin of MR diffusion signals may reveal changes that precede pathophysiological changes, electrical conductivity and congenital abnormalities, as well as the viability of the cardiac tissue non-invasively.

## Purpose

In this experimental study, we investigated the contribution of the static vasculature to the observed multi-exponential diffusion of water in isolated rabbit heart by replacing the vascular space with perfluorocarbon-emulsion (PFC). We examined the effect of flow by changing the global flow rate of a modified St. Thomas' Hospital cardioplegic solution (STH).

## Methods

**Figure 1:**
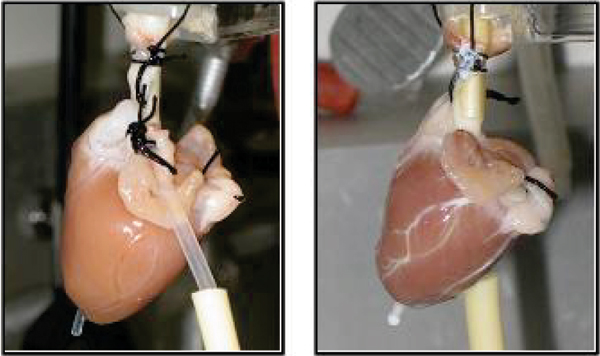
**Experimental setup of isolated heart with the aorta cannulated**. *Left*: perfused with STH, *right*: replaced with PFC emulsion. Coronary arteries shown are filled with the emulsion.

**Figure 2:**
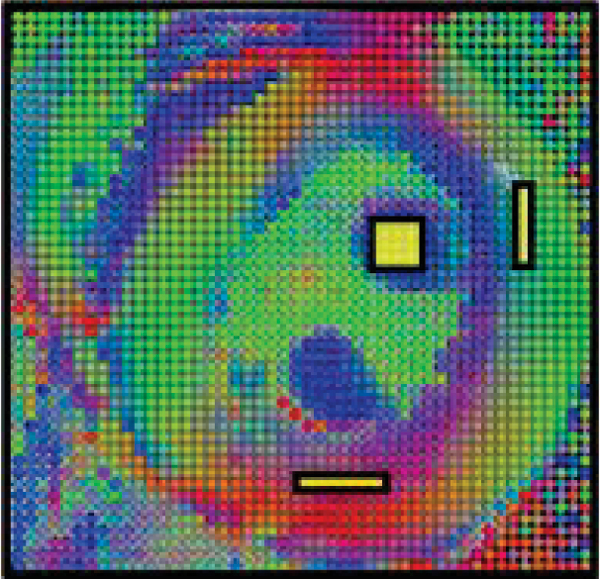
**Manual selection of ROIs**. (diffusion weighted: left to right). *Right*: free wall 1, *bottom*: free wall 2, and *middle*: papillary muscle.

Isolated perfused hearts (n = 9) of New Zealand White male rabbits (2–4 kg) were prepared according to the approved animal protocol by the UF Institutional Animal Care and Use Committee [2]. The hearts were arrested prior to imaging by switching perfusate to the STH. MR experiments were performed on an 11.1 T/40 cm clear bore magnet (Magnex Instrument Inc. UK, Bruker Instrument console). Diffusion weighted images were acquired by applying the gradients to give 14 b values up to 6500 s/mm^2^ in 6 directions with a spin echo sequence. Imaging parameters were TR = 1.5 s, TE = 27.3 ms, NA = 1, Δ = 11.59 ms, δ = 3.8 ms. Slice thickness was 2 mm with an in-plane resolution of 0.5 × 0.5 mm^2^. Five hearts underwent substitution of the STH in the coronary vascular space with 15 cc of PFC and the DTI measurement repeated (Fig. 1). Four hearts underwent variation of flow from 0 to 5 mL/min and diffusion imaging was repeated after each flow rate change. Pixel by pixel analysis was conducted on a map of the primary eigen vectors of diffusion tensor using FLTView™ (^©^2007, Barmpoutis) (Fig 2). The normalized signal intensity with b values within the manually selected ROIs was plotted to derive apparent diffusion coefficients (ADCs).

## Results

### Replacement with the PFC

**Figure 3:**
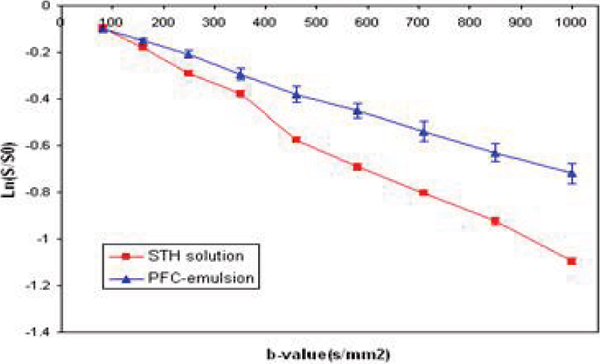
**representative logarithmic normalized signal attenuation of STH and PFC**. Each point corresponds to the mean and SD of selected ROIs.

When the vascular space was replaced with PFC particles (~Ø 450 nm), ADC decreased significantly by 23.1 ± 8.5% compared to initial measurements (p < 0.01) as b-values approached 1000 s/mm^2^ (Fig. 3). Based on the previous reports that the PFC emulsion stays inside vasculature when administered intravenously, this result suggests that the ADC decrease mostly came from the vascular replacement of STH with PFC, whose isotropic ADCs were 2.23 ± 0.24 × 10^-3^ mm^2^/s, and 1.39 ± 0.15 × 10^-3^ mm^2^/s, respectively.

### Change of vascular flow rate

**Figure 4:**
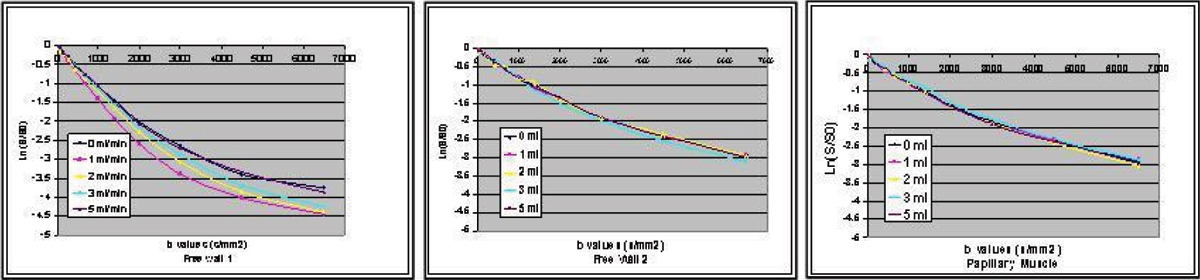
**Logarithmic normalized signal attenuation with changes in perfusate flow**. Free wall 1 *(left panel)* is parallel to the diffusion encoded direction. On the other hand, Free wall 2 *(middle panel*) and Papillary muscle *(right panel)* is oriented orthogonal to the diffusion encoding direction.

Figure 4 demonstrates the influence of flow upon the bi-exponential decline in signal intensity plotted as a function of b values in the selected ROIs. Free wall 1 (where fibers are parallel to diffusion encoded direction) shows the significant changes in the ADC over the b-values with flow change, especially 1 to 2 ml/min. Those appear to correlate with relative change in the two water pools (fast and slow components), which results from changes in the size of the tissue compartments (extracellular/intracellular). On the other hand, free wall 2 and papillary muscle don't exhibit the same degree of change in ADC or contribution of the slow component of diffusion.

## Conclusion

This study demonstrates a significant contribution of vascular compartment to the myocardial MR diffusion characteristics. First, vascular compartment appears to be a significant contributor to the fast component of MR water diffusion. Second, attenuation of signal intensity with b values up to 6500 s/mm^2^ appears to be modulated by vascular flow.

